# The Teachings of Failure

**Published:** 1892-12

**Authors:** E. Markham Skerritt

**Affiliations:** Lecturer on Medicine in the Medical School of University College, Bristol; Senior Physician to the Bristol General Hospital


THE BRISTOL

Medico-Chirurgical Journal.

DECEMBER, 1892.

### THE TEACHINGS OF FAILURE.

The Presidential Address, delivered on October 12th. 1892, at the opening of the 19th Session of the Bristol Medico-Chirurgical Society.

BY

E. Markham Skerritt, B.A., M.D. Lond., F.R.C.P.;

*Lecturer on Medicine in the Medical School of University College, Bristol;*

*Senior Physician to the Bristol General Hospital.*

My first duty is to thank you sincerely for the honour which you have done me in calling me to the presidential chair of this Society. If I do not proceed to enlarge upon my unfitness for a position of such responsibility, it is not that I am not conscious of it; but you are already too familiar with the expression of the sense of self-distrust which appears to be inseparable from the assumption of presidential cares, and almost year by year is owned to from this chair.

The past session has been one of quiet prosperity to the Society, and of freedom from war's alarms. The present year opens under circumstances of good omen. It is intended that the ordinary meetings of the session shall be held in the Medical Wing of University College, Bristol, the handsome building hard by, which, after years of anxious waiting and of hope deferred, has at length taken so satisfactory material form. I trust that it is destined to be the permanent home of the Bristol Medico-Chirurgical Society. A joint committee from your committee and the faculty of the Medical School has already been appointed to consider a scheme whereby the fine hall set apart for a library may become your recognised place of meeting, and, of more importance still, the local habitation of your library. So vigorous has been the growth of the latter, under the fostering care of your honorary librarian, that it has already all but expanded to the limits of its present abode; and I look forward with confidence to the time, in the not far distant future, when in the adjacent building there will exist a library which will bear evidence to the vitality of this Society and the activity of medical education in this city, and form a collection of works on medical and allied subjects such as will make it a boon to the members of the profession in this district, and give it undisputed position as the Medical Reference Library of the West of England.[1]

The choice of a subject for an address on such an occasion as the present is always a matter of difficulty. The history of the Society and of the life of medical institutions and medical men in this city, has again and again been recounted by those who are far more com-

[1] Since the above was written a scheme has been agreed to by the Medical School and the Society for the amalgamation of the libraries of the two institutions in the new Medical School building.

petent to deal with it than myself. A discourse on some limited subject in practical medicine or in pathology would find a more fitting place in one of the later meetings of the session. No recent great advance in our knowledge of disease, or in our ability to meet it, demands our notice. But if we look back upon the medical history of the immediate past, two episodes stand out in bold relief—one, the history of tuberculin; the other, the onslaught of influenza.

I hasten to allay the alarm which my words may have raised in your minds. It is not my purpose to inflict upon you either an analysis of the results of what is known as Koch's method, or a disquisition upon influenza. The former is, I fear, a thing of the past; the latter, I trust, is so too, and upon neither can we as a scientific profession look with satisfaction. Koch led us away after a Will-o'-the-wisp; the influenza did with us what it would; and the contemplation of this has turned my thoughts towards an aspect of our work upon which it is good for our mental discipline that we should at times meditate. This Society is wont to witness the successes of its members, and their achievements in the various regions of medicine and pathology; even our clinical failures appear before us here as pathological triumphs. I ask you to look for a few minutes upon the reverse of the picture—to submit yourselves to a short course of fasting and humiliation for sins of omission and of commission; to gaze upon our difficulties and our mistakes; remembering that, of all the lessons taught by experience, few are more valuable than those obtained from duly-appreciated *failure*.

The first part of my text is Koch's announcement of a cure for tuberculosis. It may safely be affirmed that no other event of recent years has created so profound a sensation throughout the whole civilised world. Political disturbances, however grave, wars and rumours of wars, however disquieting, have their more or less limited local incidence; even a far-reaching scourge such as cholera does not at one grasp lay hold upon all the races of mankind. But here was a thing that was brought home to every people under the sun: not a country but what was the home of tuberculosis, not a community in which it had not claimed its victims; and the greatest medical scientist of the age, a man whose reputation for accurate work had hitherto been above suspicion, the discoverer of the bacillus tuberculosis itself, came forward with the confident assertion that the dread disease was no longer incurable. "Put not your trust in princes!" Bitter disappointment has followed: the prince among scientists is fallen from his high estate; and a blow has been struck at confidence in scientific methods and scientific men which will leave its mark upon our profession for a generation.

Where was the mistake? We as a profession—against our judgment, be it said—trusted too much to the *man,* although he appeared in the novel rôle of a clinical physician. Probably all recognised the anomaly in Koch's explanation of the phenomena involved, and found it hard to understand, if tuberculin destroyed the surrounding tissue rather than the active focus itself, how the disease was to be eradicated. And the result proved that this doubt was well founded. Had the announcement come from almost any other source it would have been received far differently; but on such respected authority careful trial was imperative. What followed is matter of too recent history to need recapitulation.

When the usual methods of scientific investigation were brought to bear, evidence rapidly accumulated adverse to the method, and in its original form it is now practically abandoned. Whether any modification may ultimately be of value remains to be proved.

A special regret attaches to the recognition of Koch's own source of error. The man of world-wide scientific reputation fell into one of the commonest errors of the unscientific—he based his conclusions upon imperfect and insufficient observation.

As a side issue, it is of interest to note how any new path that is opened up tends to lead to fresh discoveries. The reaction of many tubercular subjects to tuberculin is beyond doubt, and there is much evidence to prove that when tuberculosis does not exist, reaction is comparatively rare. The fluid hence has considerable diagnostic value, though to what actual extent is still undecided. Working on the same lines, a product of the bacillus of glanders, which has been called "mallein," has been employed for the diagnosis of glanders and farcy. The result so far obtained appears to be curiously on all fours with that met with in tuberculosis. Observations have shown that, as with tuberculin in tuberculosis, so with mallein in glanders and farcy, the reaction is generally diagnostic, but not absolutely, inasmuch as it has occurred in animals free from the disease. But this is a digression.

The modern therapeutic history of tuberculosis is a record of a series of false hopes with their consequent disappointments. It is melancholy to look back upon the last few years, and see how remedy after remedy has been introduced with flourish of trumpets, has failed to stand a wider test, and has fallen into the background. Let me remind you of a few of the army of specifics:

Iodoform, aniline, thymol, tannin, ozone, calomel and corrosive sublimate, homeriana, hydrofluoric acid; antiseptic inhalations of carbolic acid, creasote, iodine, eucalyptol, iodoform, turpentine, sulphurous acid, chlorine, sulphuretted hydrogen (which were to go to the root of the whole matter by attacking the bacillus in its stronghold); rectal injections of sulphuretted hydrogen; subcutaneous and intra-tracheal injections of drugs that are much better introduced by the channels that Nature has provided; intra-pulmonary injections of iodine, iodoform, creasote, carbolic acid, iodol, camphor-carbolate, biniodide of mercury, naphthol, boronaphthol, camphor, guaiacol, and the like (designed to act directly upon the tissues into which they were injected, regardless of the impossibility of absolutely determining the diseased areas by anything short of a *post-mortem* examination); the introduction of the bacterium termo in its legions, to war upon its natural foe, the bacillus tuberculosis; Liebreich's injections of cantharidinate of potash in imitation of Koch; subcutaneous injections of goat's blood and of dog's serum;—a long array, with Koch's tuberculin at one extreme and Brown-Séquard's fluid at the other.

Even about our latest favourites, creasote and guaiacol, which still hold their ground, we are becoming uncertain. Hitherto it has been generally accepted that it is important to push the dose until large quantities are being taken daily; now we are told, in the last number of this Society's *Journal*, that large doses have no advantage over small.

Three years appears to be a fair average life time for a new specific for tuberculosis. The first year comes the announcement of the discovery, with details incontestably proving its value; the second sees a wider trial of the remedy, with varying results; the third year brings its condemnation. If we take as an illustration Weigert's hot-air treatment of phthisis; introduced in 1888, backed up by cases proving its "remarkable general and local good effects;" in 1889 well spoken of by some observers and disparaged by others; we find in 1890 the record that "the hot-air treatment of phthisis has met with nothing but discredit during the past year." And then it is pointed out that nothing else could be expected, inasmuch as both the theory and the practice are wrong: the bacillus tuberculosis does not necessarily die when the temperature of the body is raised, and indeed finds a congenial *habitat* in certain animals whose normal temperature ranges from 102.5° to 108°; and further, the inhalation of the hottest air that can be borne does not, as a matter of fact, cause more than a trifling rise in the temperature of the lung.

Then there was Bergeon's method of rectal injections of sulphuretted hydrogen, brought forward in 1886 with proof of its great value "not only in incipient but in confirmed cases of phthisis"; well-spoken of by good authorities in 1887; in the following year it is put aside, and Forchheimer relates "control" observations to prove that the result is practically the same whether the injection consists of sulphuretted hydrogen or of simple atmospheric air.

It is impossible not to be struck with the uniformity of the good results claimed for each and all of the different modes of treatment on their introduction: diminution of cough and sputum, of fever and night-sweats, gain in weight and strength, and often lessening of physical signs.

Now these are evidences of improvement that can be more or less accurately measured; and there is no doubt that they have actually occurred under all these varied therapeutic measures. Why then have they not been confirmed by a more extended experience? It would appear that a case of phthisis when taken in hand and placed under almost any treatment (provided that it is not actively harmful) tends to improve; and this view is borne out by the experience of hospitals where tuberculosis has been largely treated on general rather than specific lines. Doubtless this is in great measure due to the associated improvement in the conditions under which the patient is placed. We have also, however, to reckon with the well-known variability in the course of the disease, apart from any special treatment—its tendency to exacerbations and remissions as fresh areas of lung tissue become the seat of active mischief, which in its turn may quiet down. One thing is clear—that the good results are not limited to and do not uniformly follow the use of any one line of treatment. And we are reluctantly reminded of Sir Thomas Watson's dictum with regard to acute rheumatism: "You may be sure, when men's opinions concerning the treatment of a disease which is of common occurrence and easy recognition are thus unsettled and diverse; you may be sure, first, that no specific for that disease has yet been discovered, and secondly, that the disease is not very obedient, or not steadily obedient, to any remedial plan." Forty years after this was written, salicin and the salicylates had made it no longer applicable to rheumatic fever; let us still live in hope that the day may come when it will not be true of tuberculosis.

If our relation as a profession to tuberculosis cannot be regarded with any degree of satisfaction, the contemplation of the influenza epidemic is still less calculated to flatter our self-conceit. I mean as regards our claim to be scientific observers and therapeutists; for there is no reason to be ashamed of the self-sacrificing energy and endurance with which the foe was encountered by the medical profession.

When influenza first declared its type, when it became evident that, although severe, its paroxysm was very short, and that all the acute phenomena rapidly disappeared in a large proportion of cases, I ventured to predict that the epidemic would make the fortune of many a specific; inasmuch as the attack would rapidly pass on to its natural termination in recovery, whatever the treatment adopted—always provided that the remedial measures were not too heroic. What does a survey of the voluminous literature of the epoch reveal? A host of authorities, each of whom is fully convinced of the power of his own favourite remedy, and equally sure that his neighbour's line of treatment is most prejudicial. One writer describes his unvarying success with carbonate of ammonia and the like, and goes on to say: "As for antipyrin and its congeners, most of the deaths in the epidemic are, I think, to be attributed to their use." He is immediately followed by another, who treats 400 cases with antipyrin combined with salicylate of soda, and says: "I also am happy to say that I did not lose a single case, and found no depressing or other evil effects from the antipyrin, but on the contrary marked benefit and saving of strength." And these two are promptly discounted by a third, who testifies as follows: "I am happy to say that I did not lose a single patient from the epidemic or its sequelæ; and I used very little medicine, and that of the simplest description." The last observer, however, damages his otherwise sound scientific position by proceeding to hold forth against antipyrin, a remedy which on his own showing he had never tried.

Aconite, salicin and the salicylates, antipyrin, antifebrin, phenacetin and exalgine, carbonate of ammonia and sal volatile, iron, quinine, ergot, digitalis, camphor (recommended both for its efficacy and for its cheapness), iodide of potassium, vaccination, bromides, morphia, calomel, halviva, sulphurous acid, pilocarpine, periodate crystals, the Turkish bath, ipecacuanha, belladonna, eucalyptus, naphthol, bicarbonate of potash, electricity, and a host of other remedies—each of them found at least one believer. "I secured convalescence in 24 hours," says one observer, with a most enviable sense of power; "I find that the disease can be cut short, as easily as an ordinary bronchial attack, by the following combination"—and then comes a prescription containing ether, iron, ipecacuanha, and opium. Another relies upon hot water: drink it freely, gargle the throat with it, keep the hands in it, syringe the nose with it. Another directs that "to prevent the poisonous products of the putrefactive decomposition of food from passing into the blood, *the whole contents* of the alimentary canal should be discharged as soon as possible." Another "tried apple-water, so warmly recommended by Professor Oscar Liebreich, of Berlin, but did not observe any good results beyond an unpleasant abdominal pain and sometimes diarrhœa." Another writes: "My object is to point out the beneficial action of salicin given frequently and in large doses. It seems to arrest the course of this disease as effectually as it does that of acute rheumatism, when given in the same manner." Twenty to forty grains of salicin every hour for three to six hours, and then every two hours for a day; and by that time the fever had finished its natural course and the drug got the credit: and then, to accentuate his want of knowledge of the type of the disease, he continued the medicine "after that at wider intervals for some days," as though it were rheumatic fever that he had to treat. Alcohol, too, had its advocates. The writer who recommended aconite combined it with alcohol—as well he might. Another summed up his method by the statement that he "started, kept up, and finished off with stimulants." And an instance is mentioned in which a lady who had been ordered to take daily from 10 to 12 ounces of brandy during her illness did duly start it, and kept it up, and when last heard of had *not* finished off. The Paris correspondent of *The Lancet* (Jan. 4th, 1890) relates how the French physicians had discovered that those addicted to alcohol did not take the disease (a fact, by the way, apparently peculiar to Frenchmen), and goes on to say: "The doctors have therefore recommended warm alcoholic drinks. Unfortunately, on the strength of this prescription the public have been indulging in these drinks to such an extent that in three days, including Christmas Day, no less than 1500 persons were arrested in the streets of Paris for drunkenness. Of this number, at least 1200 stated in defence that they were simply following the treatment prescribed for influenza."

Prophylactics were of course in great demand. Eucalyptus was one of the most widely patronised, and indeed was said to be "absolutely preventive"; and persons who had a few drops on a handkerchief or on a bit of blotting-paper somewhere in the house enjoyed perfect mental immunity from attack. Those who would keep on washing their conjunctivæ with a solution of boric acid were promised exemption. Quinine was a favourite: one man took it daily all through the first outbreak of the epidemic and the succeeding interval, up to the time that he succumbed to influenza in its second outbreak, when he gave it up as defective. And the law courts have recently been called upon to decide as to the prophylactic value of another infallible safeguard, the "carbolic smoke ball."

Let me remind you again of Sir Thomas Watson's dictum: "When men's opinions concerning the treatment of a disease . . . are thus unsettled and diverse, you may be sure, first, that no specific for that disease has yet been discovered, and secondly, that the disease is not very obedient . . . to any remedial plan."

No specific for influenza *has* yet been discovered: the uncomplicated disease runs its own short well-marked course practically uninfluenced by treatment, as was found by those who let it alone; and the result is equally good, whether the patient is treated with antipyrin or with camphor water. The truth of this was indeed brought home to us when influenza assumed its graver form, when it carried off its victims with almost the uncompromising rapidity of a plague. Where were the specifics then? Where was the disease with a mortality of *nil*, so tractable that a man could write of it, "I secured convalescence"? The malady was true to itself—whether benign or malignant, it recognised no specific.

We are still far too unscientific in our methods. The snares that entrapped our forefathers are still set with success for us. That old enemy of our profession, the *Post Hoc Propter Hoc* fallacy, has renewed his youth in these days of therapeutic activity; and we are just as ready as ever to follow his lead, and be it in influenza, or in tuberculosis, or in other conditions, too prone to see cause and effect where no such relationship exists. Medicine is an inductive science, and our inductive method is too often faulty. We are in too great haste to generalise; we establish our general propositions upon too few particulars. We give a remedy in a certain number of instances of a disease, and a certain result follows, which we claim as due to the drug. A wider experience does not bear out our conclusions, and we therefore know that our premisses were wrong—that the particular propositions upon which we generalised were instances, not of cause and effect, but of accidental association. We should have waited for more experience, we should have made "control" observations, so as with greater certainty to eliminate the element of chance.

And of this I am sure, that we do not allow enough for the *vis medicatrix naturæ*. I have always held that regular medicine owes much to homœopathy, which with its infinitesimal doses has proved how recovery, especially from acute disease, will take place under what is practically no medicinal treatment whatever. A short time since I was discussing with my clinical clerks a fairly severe case of acute pneumonia; and to my query, "What would you do for him?" one of them replied, "I should not give him anything." Upon this prescription the patient made a perfect recovery. That student had learnt an important lesson—how to let well alone.

Illustrations of the difficulty of determining the action of drugs might easily be multiplied—let one suffice. When salicin and the salicylates were first brought forward in the treatment of acute rheumatism, some half-dozen similar cases of the disease were under my care in the Bristol General Hospital. Half of them were treated with salicin, and made an unusually rapid and complete recovery; the other half had practically no treatment, and ran an exactly similar course. If all had had the new remedy, it would have gained undeserved credit; but the "control" observations prevented this mistake. Note, however, that a conclusion from these instances against the value of salicin would have been equally erroneous; all that could be said was that the case for it was "not proven," and that further observation was needed. An extreme instance of the opposite method was afforded by a practitioner who recorded his first case of influenza, which happened to be in a man; after generalising on the effects of treatment, he proceeded to draw from his single case the deduction that the disease more often attacked men than women.

The problems with which we have to deal are so complex that it is little wonder if we fall again and again into error. Bacon, in his discourse on *The Advancement of Learning*, is impressed with the difficulties attending the healing art. "This much," he says, "is evidently true, that of all substances which nature hath produced, man's body is the most extremely compounded. For we see herbs and plants are nourished by earth and water, beasts for the most part by herbs and fruits; man by the flesh of beasts, birds, fishes, herbs, grains, fruits, water, and the manifold alterations, dressings, and preparations of the several bodies, before they come to be his food and aliment. Add hereunto that beasts have a more simple order of life, and less change of affections to work upon their bodies; whereas man in his mansion, sleep, exercise, passion, hath infinite variations; and it cannot be denied but that the body of man of all other things is of the most compounded mass. . . . This variable composition of man's body hath made it an instrument easy to distemper; and therefore the poets did well to conjoin music and medicine in Apollo, because the office of medicine is but to tune this curious harp of man's body and reduce it to harmony. So then the subject being so variable hath made the art by consequence more conjectural; and the art being conjectural hath made so much the more place to be left for imposture. For almost all the other arts and sciences are judged by acts, or masterpieces, as I may term them, and not by successes and events. The lawyer is judged by the virtue of his pleading, and not by the issue of the cause; the master of the ship is judged by the directing his course aright, and not by the fortune of the voyage; but the physician . . . hath no particular acts demonstrative of his ability, but is judged mostly by the event; which is ever but as it is taken; for who can tell, if a patient die or recover . . . whether it be by art or accident? And therefore many times the impostor is prized and the man of virtue taxed. Nay, we see the weakness and credulity of men is such, as they will often prefer a mountebank[1] or witch before a learned physician. . . . And what followeth? Even this; that physicians lay to themselves as Salomon expresseth it upon an higher occasion, 'If it befall to me as befalleth to the fools, why should I labour to be more wise?' . . . And doubtless upon this ground, that they find that mediocrity and excellency in their art maketh no difference in profit or reputation towards their fortune; for the weakness of patients, and sweetness of life, and nature

[1] From *montambanco*, one who mounts a bench and proclaims the virtue of his remedies—hence a quack doctor.

of hope, maketh men depend upon physicians with all their defects."

Well is it for the physician that he is still in demand, in spite of all his defects. For though since Bacon's time medicine has emerged from mediæval darkness and become a science, yet the human mind which has to face its ever-varying problems is the same, and its innate sources of error are unchanged. "For," says Bacon, "the mind of man is far from the nature of a clear and equal glass, wherein the beams of things should reflect according to their true incidence; nay, it is rather like an enchanted glass, full of superstition and imposture if it be not delivered and reduced. . . . Although our persons live in the view of heaven, yet our spirits are included in the caves of our own complexions and customs, which minister unto us infinite errors and vain opinions."

I would not have you mistake my position. I am not a sceptic as regards treatment. Fortunately for ourselves and our patients, we possess, and are from time to time adding to, a large armamentary of drugs and of remedial measures that have made good their claim upon our confidence; and modern research into the conditions of bacterial life has revealed a glimpse of a region of therapeutics whose limits it is impossible yet to define. But I do regret to see the continual series of new remedies that are introduced but to be thrown aside; I do protest against our constant abuse of the inductive method, our want of care in testing the validity of the particular propositions upon which we found our generalisations. To experimental therapeutics might well be applied the ancient saying: "The sinews of wisdom are slowness of belief and distrust."

In no department of our art are we free from risk of error and failure. Diagnosis, prognosis, treatment—I fear that there is no one of us who can truly aver that he has not been repeatedly at fault in each of these. Were I myself to go into the confessional, to own to my many known shortcomings—the unknown I shrink from picturing—I might well be held to be unworthy of the honourable position that I occupy to-night. And consciousness of failure in ourselves should make us "wondrous kind" towards the shortcomings of others.

Looked at from the standpoint of responsibility, our mistakes fall into one of two categories—the avoidable and the unavoidable; for undoubtedly no moral responsibility attaches to many of our failures. The degree to which error is unavoidable varies with the individual—varies with the mould in which the mind is cast, varies with the acuteness of vision, the sharpness of hearing, and the delicacy of the sense of touch—and no amount of training can bring all men up to the same level in medicine any more than in other callings. But enough of this personal element.

It must be fully recognised that, with our present finite knowledge, failure is sometimes unavoidable. Diagnosis affords many instances of this. Granted that there is no special difficulty in the recognition of disease when it conforms to what we regard as its type, there is an unfortunate liability for the manifestations of any given malady to become "atypical." And the more marked the variation from type, the greater is the probability that some other morbid condition will be simulated. I have heard a physician of world-wide reputation deliver a masterly clinical lecture on a case presenting all the features of tuberculosis, and point out the certainty of a fatal ending; and I have seen his surprise when the patient did not die, but lived, and showed forth the confusion that may arise between tuberculosis and typhoid fever. Again, let me remind you of Langenbuch's case, in which the symptoms of locomotor ataxy existed, and great good resulted from the stretching of the sciatic nerves; but on the death of the patient the lesions of locomotor ataxy were found to be absent. The very mention of the strange protean condition known as hysteria will recall to everyone moments of great doubt and difficulty, when upon the solution of the problem between hysteria and some grave organic disease turned the question between life and death. And not only does hysteria simulate conditions due to structural change, but it may itself be imitated by them. I well remember in my student days a girl being brought into the casualty room in a typical hysterical state: there was a slight squint, but this did not appear of importance in the face of the other symptoms. The girl was treated by such means as the resources of a hospital casualty room and the energy of youth suggested, and was sent home seemingly much better; and a day or two later came a letter announcing her speedy death, and complaining of the treatment which she had received. An almost identical case came to my knowledge some years ago in this city. A medical man asked me to be present at an autopsy on a girl who had been recognised as purely hysterical by several eminent authorities. Here, too, there was a slight squint, which did not attract much notice. One day the girl had an upset with her friends, went into the garden in a fit of hysterical crying, and fell down dead. An enormous cerebral tumour existed.

It is important to recognise that certain functional affections of the heart may simulate grave organic disease. A patient beyond middle age comes with symptoms pointing to fatty degeneration, and the physical signs are in keeping; and the man is warned to put his house in order. The general health improves, and all the symptoms disappear; and the condition is shown to have been one of functional weakness only. My experience has made me very chary of condemning a heart as fatty unless the case has been under observation for a considerable time.

In prognosis there is a double source of error. On the one hand, it is dependent upon diagnosis, which may be at fault; and on the other, it has to deal with what is more or less an unknown quantity. If man were a machine, from which a definite amount of work could be obtained from a definite supply of fuel, the problem would be simple. But given a case of acute endocarditis, who can foretell whether there will remain a murmur and nothing more, or whether grave distortion of valve will result, with rapid failure in compensation, and early death? Some years ago I saw with a friend in the country a patient with most pronounced valvular disease and secondary implication of heart. When 20 years old he was warned to be most careful; at the age of 40 a well-known authority foretold his death within twelve months; and he lived to be 60 years of age.

The experience of such things tends to undermine the simple faith with which the young practitioner emerges from his student days; and unfortunately for the mental peace, the wider experience becomes, the more do certainties give place to probabilities. And in forming an opinion it is always wise to give due weight to possibilities as well as to probabilities, and to make full allowance for the unexpected, which happens in

18 *

medicine with even greater regularity than in other walks of life.

The philosopher Bacon advises "not to engage a man's self peremptorily in anything, though it seem not liable to accident; but ever to have a window to fly out at, or a way to retire. Following the wisdom in the ancient fable of the two frogs, which consulted when their plash was dry whither they should go; and the one moved to go down into a pit, because it was not likely the water would dry there; but the other answered, 'True; but if it do, how shall we get out again?'"

*Avoidable* error may be due either to ignorance or to carelessness. In the abstract, all mistakes come from ignorance, inasmuch as there would be no mysteries to perfect knowledge; but only in so far as any man meets with failure from lack of knowledge which with our present light he might possess, is he responsible. Without doubt, however, carelessness is by far a commoner cause of error than ignorance, especially in diagnosis. My old master, Sir William Jenner, used to preach, in the emphatic way that those who knew him will remember so well, that "more mistakes are made from not looking than from not knowing." And often this "not looking" is not from wilful neglect; but the constant watching of a certain aspect of a case tends to keep the attention averted from those features which (in Professor Lloyd Morgan's terms) are "peripheral" rather than "focal" to the mental vision. And hence it happens that when another observer comes in, with his eyes not fixed in any one direction, but turned upon each detail of the case in routine succession, he sometimes makes discoveries that would have been just as easy to the brother whom he is called to meet.

Let us consider the conclusion of the whole matter. We fail from imperfect knowledge of disease, and from faulty observation of individual cases. If we would avoid error, we must one and all study more carefully the natural history of every morbid condition, and watch more closely the features of every instance that comes before us. So shall we best be able to determine the nature of the phenomena with which we meet, and to foretell their course; so shall we best discover the real effect of remedial measures, and escape the fallacy of attributing to treatment what is in truth the outcome of the untouched disease. If failure opens our eyes to our deficiencies, if it makes us more critical, more careful to avoid unscientific methods of observation and research, if it leads us to greater diligence, thoroughness, and honesty in our work, then it will not have come to us in vain.

